# Analyzing Injury Patterns in Climbing: A Comprehensive Study of Risk Factors

**DOI:** 10.3390/sports12020061

**Published:** 2024-02-19

**Authors:** Markéta Kovářová, Petr Pyszko, Kateřina Kikalová

**Affiliations:** 1 Department of Anatomy, Faculty of Medicine and Dentistry, Palacky University Olomouc, Hnevotinska 3, 77515 Olomouc, Czech Republic; marketa.kovarova04@upol.cz (M.K.); katerina.kikalova@upol.cz (K.K.); 2 Department of Biology and Ecology, Faculty of Science, University of Ostrava, Chittussiho 10, 71000 Ostrava, Czech Republic

**Keywords:** sport injuries, risk factors, injury severity, climbing phase, rock climbing, indoor climbing, bouldering

## Abstract

Climbing, a sport with increasing popularity, poses diverse risks and injury patterns across its various disciplines. This study evaluates the incidence and nature of climbing-related injuries, focusing on how different disciplines and climbers’ personal characteristics affect these injuries. Data on injury incidence, severity, and consequences, as well as climbers’ personal attributes, were collected through a questionnaire and analyzed using generalized linear models and generalized linear mixed models, Cochran–Armitage tests, and multivariate analysis. Our findings indicate a direct correlation between time spent on bouldering and lead climbing and increased injury frequency, while injury incidence decreases with time in traditional climbing. Interestingly, personal characteristics showed no significant impact on injury incidence or severity. However, distinct patterns emerged in individual disciplines regarding the recent injuries in which age and weight of climbers play a role. While the phase of occurrence and duration of consequences show no significant variation across disciplines, the intensity of the required treatment and causes of injury differ. This research provides insights into climbing injuries’ complex nature, highlighting the need for tailored preventive strategies across climbing disciplines. It underscores the necessity for further investigation into the factors contributing to climbing injuries, advocating for more targeted injury prevention and safety measures in this evolving sport.

## 1. Introduction

Climbing as a sport emerged in the 19th century, with its initial development occurring in the 1950s, particularly in England and Italy [1]. In the 1970s and 1980s, there was a significant increase in interest, with increasingly technical and overhanging routes being climbed [2]. In recent decades, the popularity of climbing has grown worldwide [1,3,4], with an estimated 9 million people engaged in the activity in the USA alone across various ages and abilities [5,6]. The rise in the number of climbers is attributed to the easier accessibility of climbing venues that have gradually been established [7]. This trend is likely supported by climbing becoming an Olympic sport [8]. In the Czech Republic, the popularity of climbing is also increasing due to the successes of some Czech climbers, such as Adam Ondra [9].

Climbing encompasses many disciplines. Bouldering, which involves climbing without a harness at relatively low heights above the ground where the climber falls or jumps onto a crash pad, is widely practiced. Over time, bouldering has become increasingly acrobatic, requiring more strength and gymnastic coordination. It is sometimes referred to as vertical parkour [10,11]. Another discipline is indoor wall climbing, where climbers use pre-placed protection on artificial walls and secure each other. Climbers either ascend by progressively placing the rope into prepared anchors or climb top rope (with the rope prepared at an anchor at the ceiling and belayed like a fishing rod) [12]. Lead climbing is similar to indoor wall climbing in that the protection is pre-placed in the rock (equipped rock). However, the spacing between protections can be greater, and the first protection is sometimes high above the ground [13]. The Czech Republic also has its unique discipline of sandstone climbing, where protection spacings are significant, primarily secured with slings, and the classifications reflect historical development rather than the actual state [14]. In traditional climbing, also conducted outdoors, climbers secure their routes using various devices (friends, stoppers, slings, etc.), which the second climber removes [13]. A specific discipline is ice climbing, involving ascending frozen waterfalls using crampons and ice axes [15]. High-altitude climbing, ranging from 4000 to 8000 m, is also a part of climbing disciplines [16,17].

Climbing is considered a relatively safe sport, yet in recent years the number and severity of climbing injuries have increased, likely due to the growing popularity of this sport [18]. Climbing carries a range of risks. It is reported that 30% to 50% of sport climbers have experienced some type of climbing injury [19,20] 44% had overuse injury, when 19% suffered overuse injury on multiple sites [12]. Each climbing discipline has its specific hazards. Injury frequency varies among disciplines. During the World Cup, there was an average of 0.74 injuries per 1000 h of climbing indoor, with bouldering at 1.47 and rope climbing at 0.29 injuries per 1000 h. Interestingly, no injuries were reported in speed climbing [21]. In the general population, 4.2 injuries per 1000 h of climbing were found, 93% injuries are overuse injuries [19]. Injury rate is about 0.2 per 1000 h if overuse injuries are excluded [5]. Climbing activities also carry the potential for fatalities, with a rate of 0.13 per 1000 climbing hours [22] and hospital mortality is extremely rare, it is less than 1% [7]. The Czech Mountaineering Association reports an average of 3.43 fatal injuries per year for 18,000 members [23].

During climbing, limb injuries are probably the most common [24]. For the upper limb, typical injuries include tendon sheath inflammations, finger swellings, tendon pulley ruptures, nerve compressions, and tendon strains. Also common are forearm flexor overloads or lumbrical syndrome, as well as partial ruptures of lumbrical muscles, primarily due to climbing with a closed grip and using finger pocket holds on one or two fingers [25]. Up to 80% of climbers experience foot pain due to ill-fitting shoes [3]. Up to 70% of cases present chronic changes in the foot, especially claw toes, hallux valgus, and changes in the skin and nails [19,26]. Recent studies underscore the significance of knee injuries in climbing, with mechanisms like high steps, drop knees, heel hooks, and falls contributing to conditions such as medial meniscal tears, particularly in indoor bouldering [27]. Individual disciplines may differ in injury composition. Boulderers have a higher frequency of lower limb injuries caused by impacts on or off the crash pad [28,29]. Common injuries here include sprains, dislocations, ankle fractures, ligament injuries, anterior cruciate ligament ruptures, or quadriceps injuries [6,19,21]. Outdoor climbers, whether on sport or traditional routes, tend to have more chronic injuries than indoor wall climbers, attributed to continuing activity despite pain. Here too, upper limb injuries predominate [30]. Injuries to other body parts—head, neck, chest, back, and pelvis—are less common but usually severe [24].

Prevention of injuries during climbing demands a comprehensive understanding of the factors influencing injury occurrence. Emphasizing the significance of the climbing phases, some studies identify specific stages of climbing on rocks and mountains as inherently perilous [31]. During climbing, this may include underestimating the terrain and late initiation of belaying, or human error such as in establishing belay systems and rope management. The risks continue with descent—ranging from improper belay device setup and rappelling off the end of the rope, to belayer mistakes. Finally, the studies also mention acts of nature from rockfalls and avalanches to gear failure [31,32]. In other disciplines, there are additional risks such as movement in high mountains and on glaciers. Risks include glacier crevasse falls, frostbite, acute mountain sickness (AMS), high-altitude pulmonary edema (HAPE), and high-altitude cerebral edema (HACE) [16,17,33]. Another significant factor can be the climber’s age. Among adolescent competitive climbers in Canada, the research found an injury rate of 4.44 injuries per 1000 h of climbing, and it was observed that adolescents are injured more frequently than both younger and older climbers [34]. Studies report that men in their thirties to forties are more often fatally injured [1,12,20,24]. However, it concludes that the number of injuries tends to increase more with the number of years spent climbing rather than with age [1,12,20]. Other factors may also play a role, studies observed a higher risk of injuries in climbers with higher BMI and a higher proportion of foot injuries in women, possibly due to the use of men’s climbing shoes, which are stiffer and create more pressure on the Achilles tendon [19]. However, other studies claim that the number of injuries does not differ significantly between men and women and is not influenced by BMI or age [1,12,20].

This work aims to conduct a comprehensive evaluation of the incidence and severity of injuries in climbing, considering a broad spectrum of factors simultaneously, unlike many previous studies that have focused on narrower aspects. Specifically, it seeks to assess injuries according to different climbing disciplines, the various phases of climbing, and the personal characteristics of climbers. Additionally, it intends to analyze whether different body parts are injured in distinct climbing disciplines among climbers in the general population. The primary emphasis of this study is its holistic approach, incorporating a multitude of factors including the phase of climbing (approach, climbing, descent), an element that has not been extensively examined in the previous literature. This inclusive approach sets this study apart, providing a more detailed and interconnected understanding of the factors contributing to injuries in climbing.

## 2. Materials and Methods

### 2.1. Study Design

A questionnaire was developed, focusing on the incidence and circumstances of injuries (at which phase of climbing the injury occurred), the severity and location of the injuries, duration of hospitalization, potential consequences, and the climbing discipline during which the injury occurred (Supplement S1). The opinion of the ethics committee for conducting the questionnaire survey has been obtained (Supplement S2). The severity of injuries was assessed based on the International Climbing and Mountaineering Federation (UIAA) injury severity grade [18], and adjusted according to recommendations from the Czech section [35].

The severity of injuries was rated numerically. Zero indicated no injury, 1 represented minor injuries (e.g., bruises, contusions not requiring medical assistance). Number 2 indicated moderate, non-life-threatening injuries requiring outpatient treatment (fractures without displacement, ligament ruptures, sprains). Number 3 denoted serious injuries without life threat, necessitating hospital treatment (dislocations, fractures of limbs and vertebrae, brain injuries). Numbers 4–5 represented more severe injuries (polytrauma always requiring medical intervention and survival with consequences, or cases resulting in death). Number 6 denoted immediate death [35]; however, it is clear by the nature of this study that this degree was not considered in the questionnaire.

For the localization of injuries, letters were used following the recommendations of the UIAA methodological commission, and based on recommendations from other sports [35,36,37] (Table 1).

We investigated the duration of hospitalization, ranging from minor injuries where the climber treated themselves or visited a doctor on an outpatient basis, to being hospitalized for 1–5 days or longer. Permanent consequences were also assessed, ranging from injuries without consequences, almost no consequences, with consequences, to serious consequences affecting social integration [35]. The questionnaire was supplemented with questions on the personal characteristics of the climber (age, sex, weight, and height) and the number of hours spent in specific climbing disciplines over the past year [1]. The full version of the created questionnaire is attached as Supplement S1.

Subsequently, the questionnaire was distributed on social networks in the following four strongest climbing groups in the Czech Republic: “Lezec.cz” (39,000 followers), “Lezci, horolezci, bouldristi (climbers)” (5800 members), “Sportovní lezení” (4000 members), and in the group “Climbing, lezení & bouldering—Czech Republic, Severní Morava a Slezsko” (3400 members). The questionnaire accepted responses from 15 December to 31 December 2023 and recorded 403 responses. However, some questionnaires were only partially completed, so 389 questionnaires were used for statistical analysis.

### 2.2. Statistical Analysis of Data

The data were processed using R version 4.2.2 [38]. We divided the data analysis into two parts. The first part of the questionnaire relates to the number of moderate and serious injuries per year (the minor injuries were filtered out), and their severity in relation to the number of climbing hours per year, and the personal characteristics of climbers. For this part of the analysis, all individuals were considered, utilizing generalized linear models (GLMs) and generalized linear mixed models (GLMMs) from the “lme4” library [39].

Initially, we conducted Kendall’s correlation analysis to investigate the associations between the number of hours spent in various climbing disciplines and participant parameters such as age, weight, height, and BMI. Additionally, we performed a Wilcoxon rank-sum test to examine sex differences. The *p*-values were adjusted using the False Discovery Rate (FDR) method to account for multiple hypothesis testing. The results of these statistical tests, completed with figures, are presented in the supplementary material accompanying this paper (Supplement S3 and S4). Further, we constructed a null GLM with a Poisson distribution for the number of injuries, where the model was weighted by the severity of the injuries (if there was no injury, the weight was set to one). A set of explanatory variables was selected stepwise based on the Akaike Information Criterion (AIC). The stepwise selection of explanatory variables was conducted by systematically adding or removing variables based on their AIC values to ensure an optimal balance between model complexity and goodness of fit, thereby enhancing the predictive accuracy and generalizability of our final model. Explanatory variables were the amount of time spent in individual climbing disciplines as follows: (i) in absolute terms, the number of hours per year in indoor wall climbing, lead climbing, small rocks’ climbing, traditional climbing, bouldering, ice climbing, and high-altitude climbing (4000–8000 m), along with the total time spent climbing; (ii) the relative proportions of time spent in individual climbing disciplines in relation to the total climbing time. Models accounting for absolute values and those considering relative values were compared based on AIC. In all stepwise selections, second-degree polynomials for continuous variables and two-way interactions of all explanatory variables were also considered.

In the second phase, using GLMMs with a Poisson distribution, models weighted by injury severity were created to explore the relationship between the number of injuries and the personal characteristics of climbers (sex, age, weight, height, BMI). The random terms included the nationality of the climbers (Czech Republic or Slovakia, as this variability was not our primary focus but could differ between countries due to the different range of activities available for climbers) and the total sum of climbing hours (as this essentially represented the degree of exposure to injury risk; however, this relationship might not be linear or straightforward, as would be appropriate when using an offset instead of a random term). Due to the collinearity between weight, height, and BMI, these variables were tested separately in the first phase, and their significance was determined by comparing them with the null model based on AIC. Since none of the explanatory variables proved significant, a more complex model was not constructed with them. Second-degree polynomials for quantitative variables were also considered, but since none of them were significant, they are not further mentioned.

Furthermore, only data about injuries were filtered, and using GLM and GLMM with a binomial distribution, the average severity of injuries was analyzed, this time with the model’s weight defined by the incidence of injuries. The influence of time spent in individual disciplines per year versus the influence of relative proportions of time dedicated to individual disciplines (GLMs compared using AIC) and the impact of climbers’ personal characteristics (GLMMs) were again studied.

The second part of the questionnaire focused on the climbers’ most recent injury. Here, the analysis concentrated on the presence or absence of injuries in relation to more common disciplines within the dataset: bouldering, lead climbing, indoor wall climbing, and other disciplines combined (this category included other, less frequent disciplines of climbing). In this phase of analysis, lead climbing also represented small rock climbing, due to the almost absolute overlap of respondents who reported their last injury in both disciplines. Small rock climbing, a distinct climbing discipline, is characterized by its emphasis on shorter routes, typically not exceeding one pitch, and often prioritizes technical skill and precision over endurance. This definition aims to elucidate why climbers may not distinctly categorize their activities in this discipline, thereby clarifying the observed trends in differentiation within the survey responses.

Each analysis considered only climbers for whom the possibility of a given injury was relevant. For instance, if the presence or absence of injuries during bouldering was analyzed, only individuals who had spent time bouldering in the last year were filtered. Initially, a GLM model with a binomial distribution was constructed, examining the presence or absence of the last injury from a given discipline in relation to the amount of time dedicated to that discipline. In the second phase, a GLMM with a binomial distribution was constructed, where nationality and the amount of time dedicated to this discipline in the last year were random terms, and the climbers’ personal characteristics were explanatory variables. Due to the collinearity between weight, height, and BMI, only the most significant explanatory variable was included in the model. Second-degree polynomials for quantitative variables were also considered, but since none of them were significant, they are not further mentioned. The models were evaluated using ANOVA from the “car” package [40].

Further, we focused on how injuries incurred during bouldering, lead climbing, indoor wall climbing, and in other disciplines combined differ in terms of the phase when the injury occurs, the intensity of required treatment, and the duration of consequences. For this part of the analysis, only injured individuals were filtered. Frequencies in the various categories were compared using Cochran–Armitage tests from the “coin” library [41]. We also compared how often the fault could be attributed to the belayer, the climber, or an act of nature using the Chi-squared test—from this comparison, bouldering was excluded as it is practiced without a belayer. For the same reason, the categories of rappelling and descent were combined when comparing in which phase of climbing the injury occurs.

Finally, Canonical Correspondence Analysis (CCA) in Canoco 5.01 [42] was used to examine the distribution patterns of injuries across various climbing disciplines and their association with specific body parts. The CCA analysis was complemented by a Monte Carlo permutation test with 1999 permutations to validate the results, providing a comprehensive overview of injury occurrences correlated with different climbing styles. Graphic outputs were created using the “jtools” and “ggplot2” libraries in R [43,44], and using Canoco 5.01 [42]. To ensure clear visualization and to prevent overlap of data points, a jittering technique has been applied to the plotted values in R graphical outputs. The raw data is included as a Supplement S5.

## 3. Results

### 3.1. Overall Injury Incidence in Relation to Climbing Discipline and Climber’s Personal Characteristics

When comparing the model working with the absolute amount of time dedicated to individual disciplines and the model working with relative proportions of time dedicated to individual disciplines, it was found that the model based on the absolute amount of time (df = 5, AIC = 1565.30), was better than the one based on its proportion (df = 6, AIC = 1579.91), although both were good compared to the null model (df = 1, AIC = 1604.47)

**Figure 1:**
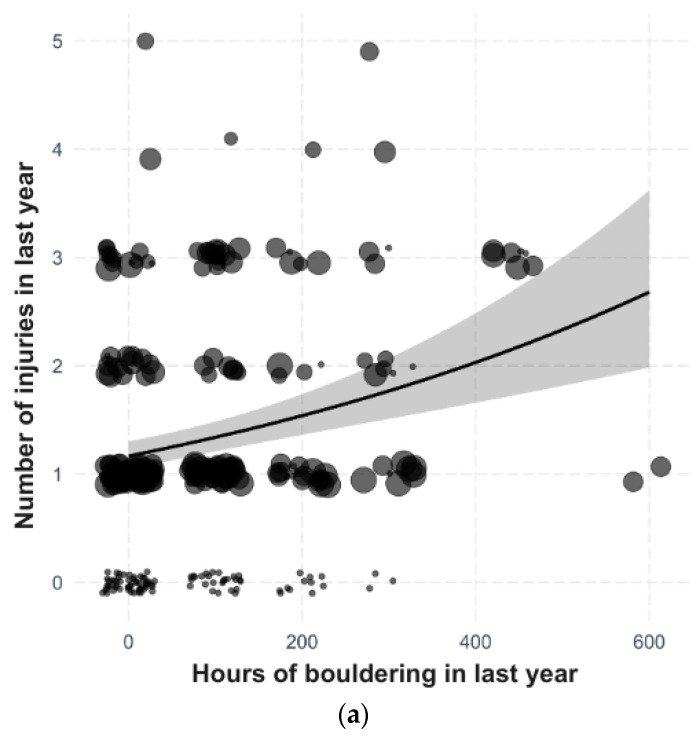

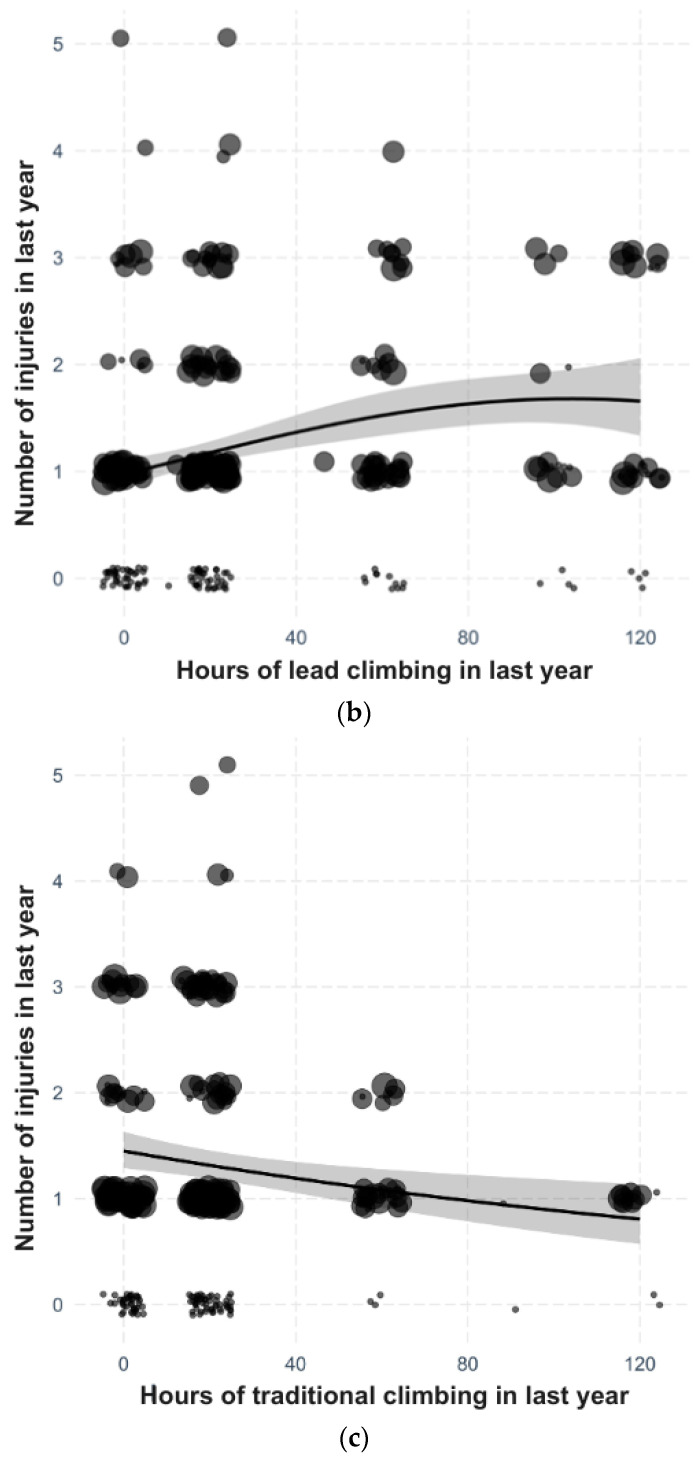
Model estimation for the number of injuries depending on the amount of time dedicated to (**a**) bouldering, (**b**) lead climbing, (**c**) traditional climbing (mean ± 95% CI), with the size of the point corresponding to the weight of the point.

The number of injuries significantly increases with the amount of time dedicated to bouldering (df = 381, χ^2^ = 24.31, *p* < 0.001, Figure 1a), and with the amount of time dedicated to lead climbing (df = 379, χ^2^ = 14.49, *p* < 0.001, Figure 1b), although here for very active climbers (>60 h), the number does not increase further. Conversely, the number of injuries significantly decreases with the amount of time dedicated to traditional climbing (df = 378, χ^2^ = 9.38, *p* = 0.002, Figure 1c). Other types of climbing did not have a significant effect (*p* ≥ 0.05) nor did they improve the model in terms of AIC.

None of the personal characteristics of the climber have a significant impact on the number of injuries: neither sex (df = 379, χ^2^ = 1.03, *p* = 0.311), age (df = 379, χ^2^ = 0.33, *p* = 0.565), weight (df = 379, χ^2^ = 1.72, *p* = 0.190), height (df = 379, χ^2^ = 1.94, *p* = 0.164), nor BMI (df = 379, χ^2^ = 0.75, *p* = 0.387).

### 3.2. Overall Injury Severity in Relation to Climbing Discipline and Climber’s Personal Characteristics

When comparing the model working with the absolute amount of time dedicated to individual disciplines and the model working with relative proportions of time dedicated to individual disciplines, it was concluded that neither of the models is better than the null model.

None of the personal characteristics of the climber had a significant impact on the severity of injuries: neither sex (df = 283, χ^2^ = 1.17, *p* = 0.279), age (df = 283, χ^2^ = 1.15, *p* = 0.284), weight (df = 283, χ^2^ = 0.78, *p* = 0.379), height (df = 283, χ^2^ = 0.78, *p* = 0.379), nor BMI (df = 283, χ^2^ = 0.07, *p* = 0.791).

### 3.3. Last Injury in Relation to Climbing Discipline and Climber’s Personal Characteristics

**Figure 2:**
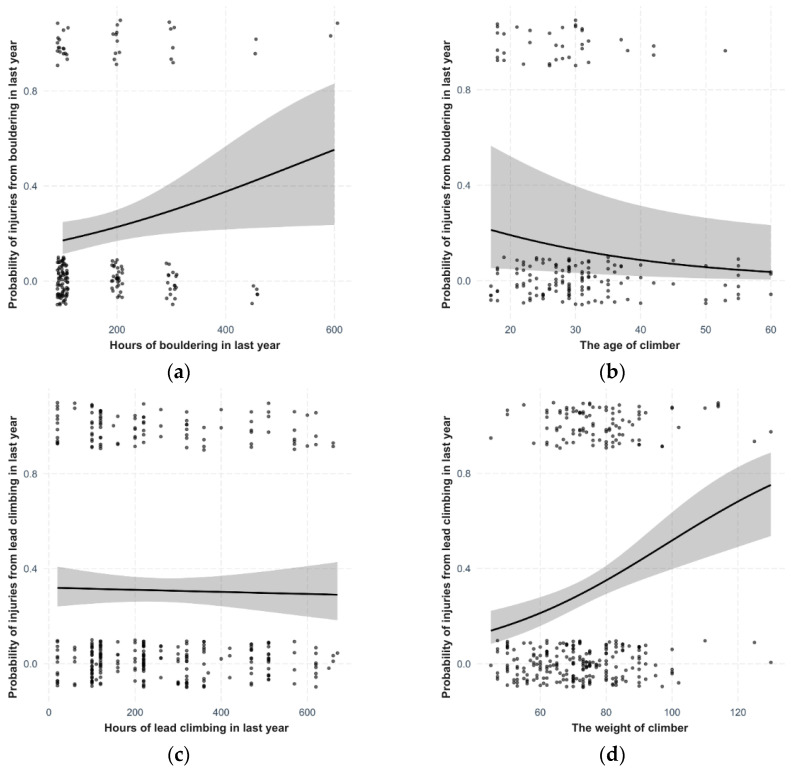

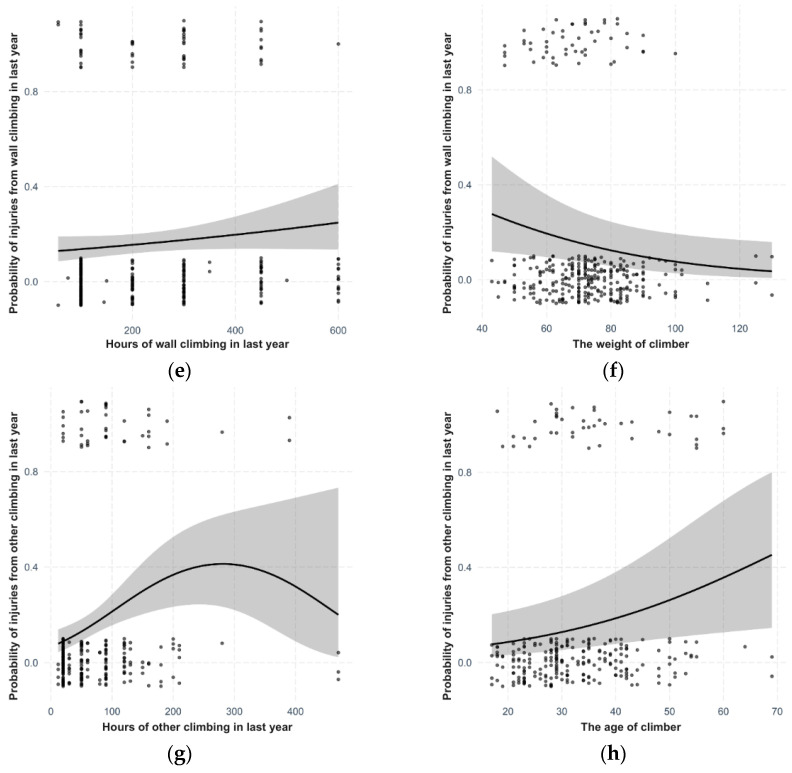
Model estimates for the probability of injury during (**a**) bouldering depending on the amount of time dedicated to bouldering, (**b**) bouldering depending on the age of the climber, (**c**) lead climbing depending on the amount of time dedicated to lead climbing, (**d**) lead climbing depending on the weight of the climber, (**e**) indoor wall climbing depending on the amount of time dedicated to indoor wall climbing, (**f**) indoor wall climbing depending on the weight of the climber, (**g**) other disciplines depending on the amount of time dedicated to other disciplines, (**h**) other disciplines depending on the age of the climber.

The probability of the last injury occurring during bouldering significantly increases with the amount of time dedicated to bouldering (df = 169, χ^2^ = 4.60, *p* = 0.001, Figure 2a). After adjusting for this influence, the probability of injury during bouldering tends to decrease with the climber’s age (df = 167, χ^2^ = 3.82, *p* = 0.051, Figure 2b). None of the other personal characteristics had a significant influence (*p* ≥ 0.05) nor did they improve the model in terms of AIC.

The probability of the last injury occurring during lead climbing does not depend on the amount of time dedicated to lead climbing (df = 329, χ^2^ = 0.09, *p* = 0.759, Figure 2c). Despite adjusting for the potential influence of this variable, the probability of injury during lead climbing strongly increases with the weight of the climber (df = 327, χ^2^ = 16.03, *p* < 0.001, Figure 2d). None of the other personal characteristics had a significant influence (*p* ≥ 0.05) nor did they improve the model in terms of AIC.

The probability of injury occurring during indoor wall climbing does not depend on the amount of time dedicated to indoor wall climbing (df = 322, χ^2^ = 2.22, *p* = 0.136, Figure 2e). Despite adjusting for its influence, the probability of injury during indoor wall climbing decreases with the weight of the climber (df = 320, χ^2^ = 4.83, *p* = 0.028, Figure 2f). None of the other personal characteristics had a significant influence (*p* ≥ 0.05) nor did they improve the model in terms of AIC.

The probability of injury occurring during other disciplines of climbing significantly increases with the amount of time dedicated to these disciplines (df = 259, χ^2^ = 12.48, *p* = 0.002, Figure 2g), but only up to about 200 h—the effect was not observed in the few climbers who climbed significantly more than this limit. After adjusting for the influence of this variable, the probability of injury during other disciplines of climbing increases with the age of the climber (df = 257, χ^2^ = 6.11, *p* = 0.013, Figure 2h). None of the other personal characteristics had a significant influence (*p* ≥ 0.05) nor did they improve the model in terms of AIC.

### 3.4. Phases of Injury Occurrence, Causes of Injury, Intensity of Required Treatment, and Duration of Consequences

**Figure 3:**
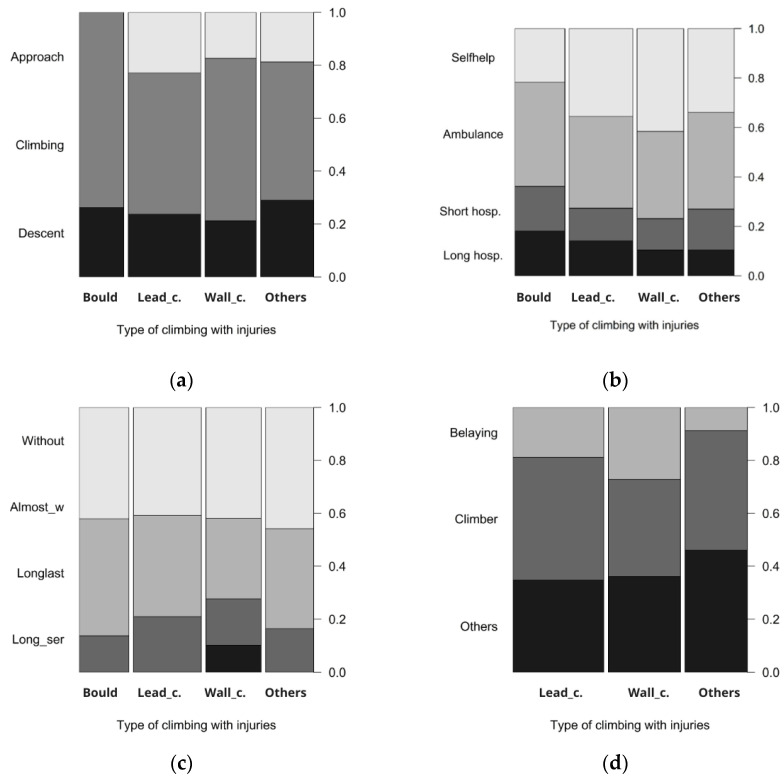
Spineplots showing proportionally for bouldering (bould), lead climbing (Lead_c), indoor wall climbing (Wall_c), and other disciplines (Others), (**a**) in which phase injuries occur in different disciplines, (**b**) the intensity of treatment required for injuries in different disciplines, (**c**) the duration of consequences caused by injuries in different disciplines (without consequences, almost without consequences, long-lasting consequences, long-lasting serious consequences), (**d**) the cause of injuries in different disciplines.

The disciplines during which the last injury occurred do not significantly differ in the phase in which the injury occurs (df = 3, χ^2^ = 3.29, *p* = 0.349, Figure 3a). The last injuries most frequently occur during the climbing itself. However, we can say that bouldering has the lowest rate of injuries at the approach, while lead climbing has the highest. Indoor wall climbing exhibited the lowest rate of injuries during descent, whereas other disciplines (less frequent) demonstrated the highest rate of injuries in this phase.

The disciplines during which the last injury occurred differ in the intensity of the required treatment (df = 3, χ^2^ = 17.16, *p* < 0.001, Figure 3b). Generally, climbers most often treat themselves or seek outpatient treatment. In the case of indoor wall climbing, generally the least intense treatment is required, with self-treatment being most common. Bouldering requires the highest intensity of treatment and also has the highest ratio of hospitalizations longer than five days.

The disciplines during which the last injury occurred do not differ in the duration of consequences (df = 3, χ^2^ = 1.54, *p* = 0.673, Figure 3c). Generally, injuries most often have no consequences or almost no consequences. We identified serious long-term consequences resulting from indoor wall climbing, with two participants.

The disciplines during which the last injury occurred differ in the reported cause of the injury (df = 4, χ^2^ = 16.38, *p* = 0.003, Figure 3d). In indoor wall climbing, the climber’s error is less frequently reported as the cause of injury than in other types of climbing. Bouldering was excluded from this comparison as there is essentially no possibility of attributing blame to a belayer.

### 3.5. Injured Body Part

**Figure 4:**
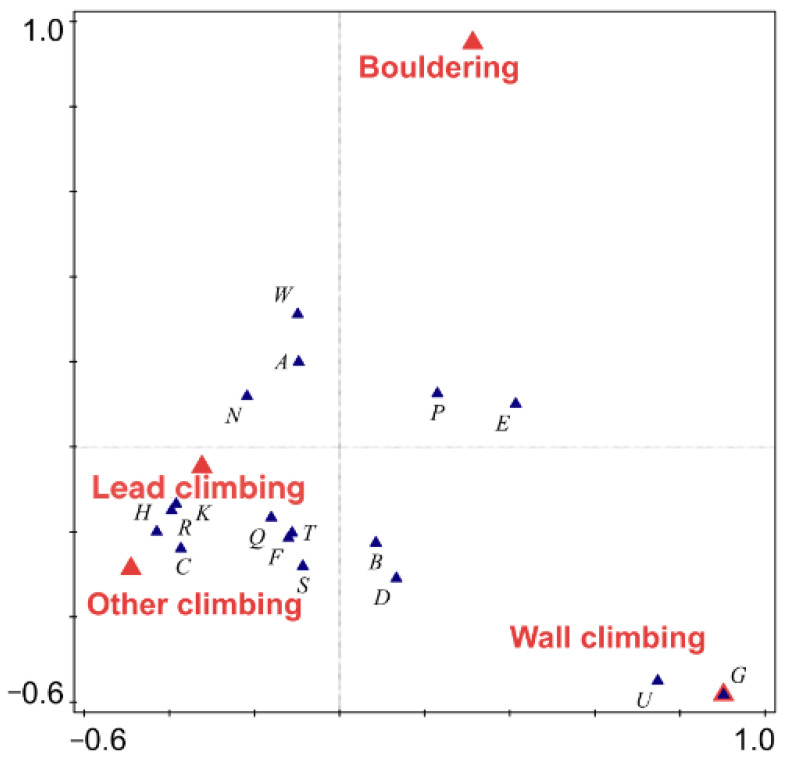
Canonical Correspondence Analysis (CCA) diagram shown on the first two axes, explaining 4.20% of the variability, the types of climbing associated with injuries to different body parts (H—head, face; N—neck, cervical spine; S—shoulder; U—upper arm; E—elbow; R—forearm; W—wrist; P—hand, finger, thumb; C—sternum, ribs; D—upper back; B—lower back; G—hip, groin; T—thigh; K—knee; Q—lower leg, Achilles tendon; A—ankle; F—foot).

Different disciplines vary in the body parts injured (pseudo-F = 4.00, *p* < 0.001, Figure 4). Injuries to the arm, hip, and back are typical for indoor wall climbing, while injuries to the head, neck, chest, shoulder, forearm, thigh, knee, shin, and Achilles tendon are typical for lead climbing and other types of climbing—both types of climbing cause approximately similar injuries. In bouldering, injuries to the hand and thumb, wrist, elbow, and ankle occur more frequently.

## 4. Discussion

### 4.1. Injury Incidence and Severity in Relation to Climbing Discipline

Regarding the climbing discipline, the number of injuries increases with the amount of time dedicated to bouldering, as depicted in Figure 1. This finding aligns with those of other studies [12,19]. Bouldering appears to be riskier in terms of injuries per 1000 h of climbing. Comparatively, boulderers had 1.47 injuries per 1000 h of climbing, whereas indoor wall climbers had only 0.29 injuries per 1000 h in study of Backe et al. [19]. However, it is important to note that injury rates can vary significantly among different studies. For instance, in lead climbing, these rates range from as low as 0.02 per 1000 h to as high as 13.04 per 1000 h [4,19,21,45], and for speed climbing, even a rate of 0.00/1000 h was observed [46]. In bouldering, it is presumed that the higher incidence of injuries may reflect the intense difficulty of modern bouldering, where climbers perform more intense athletic moves than are possible on longer routes [47]. Modern bouldering also involves more aggressively curved climbing shoes, which change the landing [10]. When bouldering outdoors, there is a higher prevalence of finger and hand ligament injuries while indoor bouldering more frequently results in lower body injuries, most commonly caused by falls or jumping off [10,11]. According to our data, the number of injuries in bouldering increases with the number of hours spent climbing, but does not continue to rise for active climbers who climb more than 60 h annually. This could be explained by the higher experience of climbers who already have sufficient strength, balance, and flexibility for climbing compared to beginners, who climb less and are particularly more prone to risks during falls, as stated by [18].

In lead climbing during lead climbing (gradually placing the rope into prepared anchors), our data show that the number of injuries increases with the number of hours spent climbing. Higher risk in lead climbing is also reported by other authors [12,32,48]. This could be due to more red point (RP) attempts [20]. The high proportion of falls during RP might be the reason why we observed a higher number of injuries in climbers with higher weight as they create a greater impact when striking the rock (the fall is longer, lifting the belayer more). Lead climbing is considered easier and safer, so climbers tend to train harder at the limits of their performance, leading to more overuse injuries (hands, ligaments) [19,28]. Overuse injuries such as ruptures and other ligament damages were also frequent in our data. Additionally, in lead climbing, there is the risk of a long fall to the ground if the first protection is highly placed [13,14,32], or a hard fall into the rope during static belaying [49]. Small rocks’ climbing has been added to the section of lead climbing; according to our data, the same climbers engaged in both disciplines, even if it may include sport routes and traditional routes. Almost all respondents who mentioned that their last injury occurred during lead climbing also specified that it happened on small rocks. They thereby ruled out injuries during multi-pitch climbing.

According to our data, the number of injuries decreases with the amount of time devoted to traditional climbing. Climbers who engage in traditional climbing tend to be significantly more technically advanced and are more likely to have chronic illnesses (possibly because they often continue climbing despite pain) than injuries [18,30,50]. We therefore assume that as climbers gain more experience and skills, the number of injuries decreases. The lower number of injuries may also be due to the style of ascent since, in traditional climbing, climbers aiming for on-sight (OS) or flash ascents encounter fewer “red pointing” attempts, which increase the risk of injury due to the high number of falls required [20]. However, research indicates a higher likelihood of fatal errors and additional risks of accidents during rappelling [32]. Due to the nature of our survey, we could not record fatal injuries and did not observe a higher number of accidents during rappelling. Nevertheless, our data suggest that injuries in traditional climbing are less frequent but generally more severe. This is often due to long falls, and research adds that these falls are particularly caused by the lack of mobile pros or their improper placement [51].

### 4.2. Overall Injury Incidence and Severity and Last Injury Occurrence in Relation to Climber’s Personal Characteristics

We did not find a significant influence of any personal characteristics of climbers on the overall number or severity of injuries: neither sex, age, weight, height, nor BMI, similar to other studies [1,12,20]. Only one study describes a correlation between higher BMI in boulderers and the frequency of injuries, due to higher forces upon impact and the resulting strain on the upper extremities [19]. Research focused on young climbers indicates higher numbers of injuries among those aged 11–14 years [4], or a higher proportion of serious injuries in the group of climbers in their thirties and forties [24]. Conversely, in older climbers, although performance-wise equally capable, degenerative changes and associated chronic injuries are more frequently observed [52,53].

However, if we focus more closely on the occurrence of the last injury in different disciplines, according to our data, the likelihood of injury in bouldering decreases with age, similar to findings from other research [4]. We assume that younger climbers have higher activity in jumps and predispositions caused by incomplete skeletal development such as epiphysial fractures [2,10,11,19,54]. In older climbers, there is likely an increase in age-related self-responsibility [54]. The probability of injury in lead climbing according to our data significantly increases with the climber’s weight, likely due to the impact with the rock during falls [20]. Moreover, heavier climbers also place more strain on their hands and ligaments, leading to overuse injuries [28]. We found that the likelihood of injury in indoor wall climbing does not depend on the amount of time spent climbing. We believe that indoor wall climbing has prepared belay systems, thus falls are usually short, and for beginners, even top rope belaying is possible, where the chance of injury is very low. Similar conclusions were reached by other studies [12,29,33]. In top rope climbing, longer falls occur only in the case of a belayer’s error [49].

The likelihood of injury in other climbing disciplines significantly increases with the amount of time dedicated to these disciplines of climbing, but this trend is not observed beyond 200 h per year. The increasing number of injuries with the amount of time is also reported in other research [26]. Among very experienced climbers, who climbed more than 200 h a year, the number of injuries did not continue to increase. It is possible that such experienced climbers are able to correctly assess situations and prevent dangers. According to our data, the number of injuries also grows with the climber’s age. The increasing number of injuries with age may be due to the influence of degenerative changes [53].

### 4.3. Phases of Injury Occurrence, Causes of Injury, Intensity of Required Treatment, Duration of Consequences

In all climbing disciplines, we found that injuries most often occur during the climbing itself, which is interesting because in some types of climbing, the descent and approach can be more time consuming than the climbing itself (this is especially typical for high-altitude climbing, but also common in ice climbing, and occasionally in traditional or lead climbing in alpine environments). This demonstrates a certain level of danger inherent in climbing itself.

According to our data, the phase during which the injury occurred does not significantly differ among the various climbing disciplines. In rope-secured climbing, the role of the belayer comes into play, who can be a cause of injury and can also be injured themselves (falling objects, impact against the wall when catching a fall, burns from the rope), as also stated in other studies [29,32,55]. In bouldering, although there is no belaying with a rope, injuries caused by improper landing on the crash pad, both on and off it, are more frequent, as confirmed by [19,21,30,56]. Moreover, the strong bend of bouldering shoes increases the risk of injury during jumps and landings [10]. Attention is also drawn to bouldering crash pads in poor condition [6,29,47]. Injuries during descent can occur, for example, due to inattention while rappelling [31]. As reasons for accidents, research and our survey indicate human factor failures, primarily loss of concentration, fatigue, material failure, acts of nature (e.g., falling rocks), or a combination of multiple factors. Similarly, another study reports ignorance or disregard of methodologies as a cause of injuries [19].

We found that the intensity of required assistance, or the length of hospitalization, is generally highest in bouldering, similar to other research [10]. Previously, injuries according to UIAA were most often classified as level 3, but today, more severe injuries are appearing, likely due to the increasing difficulty of bouldering, the need for numerous jumps, and dynamic movements [18]. In contrast, in indoor wall climbing, most injuries are of low severity, UIAA grade 1 (scratches, bruises), where climbers often treat the injuries themselves.

The duration of consequences does not differ among climbing disciplines. Generally, and also according to our data, the most common injuries are without consequences, although from a long-term perspective, there is a warning about the potential development of osteoarthritis and functional limitations and long-term pain, and polytrauma and fatal injuries occur as well [13,24,31].

In indoor wall climbing, a belayer’s error is more frequently reported as the cause of injury than in other types of climbing. Individual belay points are usually close to each other, and there is also the possibility to climb using top rope, where the belayer is one of the few factors that can influence injuries. They might give too much slack in the rope, thereby prolonging a fall, or not releasing the rope quickly enough, etc. [29]. Belayers themselves can also be injured. In our case, belayers were most often injured by rope slippage in their hands and burns from the rope, or by falling rocks in accordance with the literature [32,55]. Unlike others [31], we did not record any instances of belayers being thrown against the rock.

### 4.4. Injured Body Part

We found that different disciplines are associated with injuries to different parts of the body. The utilization of CCA illuminated the intricate relationship between different styles of climbing and injury occurrences, offering a nuanced perspective on how specific climbing disciplines are associated with particular injury patterns, thereby enriching our understanding of injury prevalence and prevention in the context of climbing. In bouldering, the most common injuries occur in the upper extremities, particularly in the hands, fingers, wrists, and elbows, as also noted in other recent studies [1]. This observation contrasts with earlier literature which emphasized lower limb injuries [28,29], suggesting a possible evolution in injury patterns or differences in climbing styles and practices. Upper extremity injuries often involve overuse during training on fingerboards and overuse of the forearm flexors, especially the brachioradialis and flexor digitorum muscles, as well as overuse or tearing of the ligaments, as similarly reported in other studies [10]. According to our data, injuries to the lower extremities frequently involve the shins and Achilles tendons, similarly to other reports [10]. Injuries to the lower extremities mainly occur during jumps and landings [1,19,21,30,56]. Risks are increased for advanced climbers by wearing small and strongly curved shoes [10,57], as such footwear increases the likelihood of injuries during landings by keeping the foot in a supinated position, thereby lengthening the peroneus longus and brevis muscles, which are important for ankle stability [58]. Wearing climbing shoes that are too small leads to chronic changes and injuries, including changes in the step and the development of transverse (and longitudinal) flat feet and in diabetics, to neuropathy [59,60,61]. It can also lead to the development of hallux valgus, which should be particularly avoided in children [62].

According to our data, the most common injuries on climbing walls are to the arms, hips, and back. Similarly, a high proportion of upper extremity injuries is reported by [28,50], and about 32% of injuries on walls involve the upper extremities [12]. Lower extremity injuries are often caused by specific climbing techniques, such as pointing, drop knee, and heel hook [5]. The literature indicates that lower extremity injuries often involve the back, in addition to affecting the hips and knees, a finding also reflected in our recorded back injuries [12,27]. The development of back problems, leading to damage in the lumbar vertebrae, is attributed to falls and the strain on the lumbar spine during belaying [6].

Common injuries in lead climbing and other types of climbing encompass a wide range of traumas. Particularly, we recorded head injuries, shoulder and collarbone injuries, hand and finger injuries, rib injuries, knee, shin, ankle, and toe injuries. Head injuries occur either from a climber’s fall or from falling objects (stones or items dropped by climbers) with only about 20% of climbers reportedly using helmets [28,55,63]. In the case of foot injuries in lead climbing, the most common are bruises, fractures of the talus and calcaneus, sprains, and ligament damage, as found in previous research [50,56,62]. Additional injuries include torn menisci and musculus quadricep injuries [6]. Chronic problems include numbness and pressure sores on the dorsal side of finger joints, dead nails, missing nails, infections, pressure sores, dermatomycoses, and subungual hematoma [56]. These foot issues are not attributed to climbing activity but to wearing small shoes [64]. In small shoes, there is supination and contraction of the toes. The interphalangeal joints are flexed, and the metatarsophalangeal joints are in hyperextension. The weight is not distributed on metatarsophalangeal joints 1–5 and the heel, but solely on the distal phalanx of the hallux [56].

A large portion of injuries recorded in lead climbing involves injuries to the upper extremity and hand, as also found by [19,28,34,50]. We frequently identified injuries to the fingers, especially the pulleys, as well as the wrist and elbow, similar to findings by [64]. Generally, the most common hand injuries include sprains, strains, tendonitis (fingers), chronic overuse (fingers, elbow), lacerations (fingers), and fractures [50]. The most common site of injury is the proximal interphalangeal (PIP) joint, PIP joint collateral ligament sprain, and injuries to the flexor digitorum superficialis and profundus tendons and tendon sheath complex. Finger pulleys are loaded up to a force of 450 N, with the A2 and A4 pulleys being the most frequently injured due to their frequent overloading and relative weakness [5,65]. Specific climbing techniques—such as one-finger pocket holds—can lead to fractures and acute lumbrical muscle strains, and chronic scarring [66]. Overuse can result in myotendinous strains, tenosynovitis, tendinopathy, medial nerve entrapment in the carpal tunnel (“carpal tunnel syndrome”) [5], early finger osteoarthritis [67], osteophytes, or e.g., epiphyseal stress fractures among younger climbers. Older, more experienced climbers had significantly more overstrain chronical injuries than acute injuries [2,5,68].

In the literature, the influence of addictive substances frequently appears as a common reason for injuries. It is reported that 20–25% of hospitalized individuals had consumed addictive substances, most commonly marijuana or alcohol [50,63]. Although climbers in our survey sometimes cited unusual situations as the cause of injuries, addictive substances did not appear as a reason. The question remains whether Czech and Slovak climbers actually climb sober, or whether they simply do not consider substances like alcohol as a cause for injury.

Climbing is still considered a safe sport, but in recent years, the number of accidents has been increasing [18]. Moreover, the exact number of injuries is difficult to determine and document because only a small percentage of climbers participate in research or diligently record their injuries in accident reports. Some injuries are considered minor—such as sprains, strains, tendon stretches—and climbers continue to train with these injuries, disregarding the need for recovery. According to the literature, only 36% of the injured seek medical help [28]. Thus, the question remains how many climbers take injuries lightly, even though a doctor’s visit could significantly improve their condition. There is particularly a need for education among climbers to make them aware of the risks they are taking, to encourage them to record their injuries and to follow methodological guidelines.

Further detailed research would also be valuable on how climbing shoes affect the rate of injuries. Research attributes an increased risk of injuries upon impact and the development of chronic diseases to climbing shoes [10,62]. However, measuring the actual pressure and forces exerted while climbing in climbing shoes is a matter of advanced measuring equipment.

## 5. Conclusions

Our study unveils consistent injury patterns across various climbing disciplines. Bouldering stands out for its higher incidence of upper extremity injuries, particularly affecting the hands, fingers, wrists, and elbows. In lead climbing, a wider array of injuries is observed, ranging from head injuries and shoulder or collarbone injuries to foot-related issues, often associated with the use of climbing shoes. Traditional climbing, while demonstrating a lower overall injury incidence, tends to result in more severe injuries, frequently involving long falls.

Remarkably, personal characteristics such as sex, age, weight, height, or BMI did not significantly impact the overall number or severity of injuries across climbing disciplines. Nevertheless, specific trends surfaced in the context of climbing activities, such as the correlation between increased weight and injury risk in lead climbing.

Of note is the observation that injuries most commonly occur during the climbing phase, although it is acknowledged that the approach and descent phases may be more time consuming. This underscores the inherent dangers of the activity. Human factors, including loss of concentration and fatigue, play a substantial role in accidents, emphasizing the critical importance of climber awareness and adherence to safety protocols.

## Figures and Tables

**Table 1:** Letters for injury localization.

Body Part	Letter:	Body Part	Letter:
Head/Face	H	Abdomen	O
Neck/Cervical spine	N	Lower back/pelvis/sacrum	B, L
Shoulder/Clavicle	S	Hip/groin	G
Upper arm	U	Thigh	T
Elbow	E	Knee	K
Forearm	R	Lower leg/Achilles tendon	Q, A
Wrist	W	Ankle	A
Hand/finger/thumb	P	Foot/toe	F
Sternum/ribs/upper back	C, D		

Injury localization according to UIAA [35].

## Data Availability

Data are contained within the Supplementary Material S5.

## Supplementary Materials

The following supporting information can be downloaded at: https://www.mdpi.com/article/10.3390/sports12020061/s1, S1(a,b): Questionnaire; S2: Opinion of the ethic committee; S3, S4: Supplementary Analysis: Correlations of personal characteristics of climbers with hours spent in individual climbing disciplines; Data S5: Raw data.
