# Supplementary material for: Analyzing Injury Patterns in Climbing: A Comprehensive Study of Risk Factors
Source: Sports (Basel). 2024 Feb 19;12(2):61. doi: 10.3390/sports12020061 (PMC10892067; doi:10.3390/sports12020061)
Supplement: Supplementary file 1 [file sports-12-00061-s001.zip › S3 supplenetary figures - age, weight, height, sex, BMI.pdf]

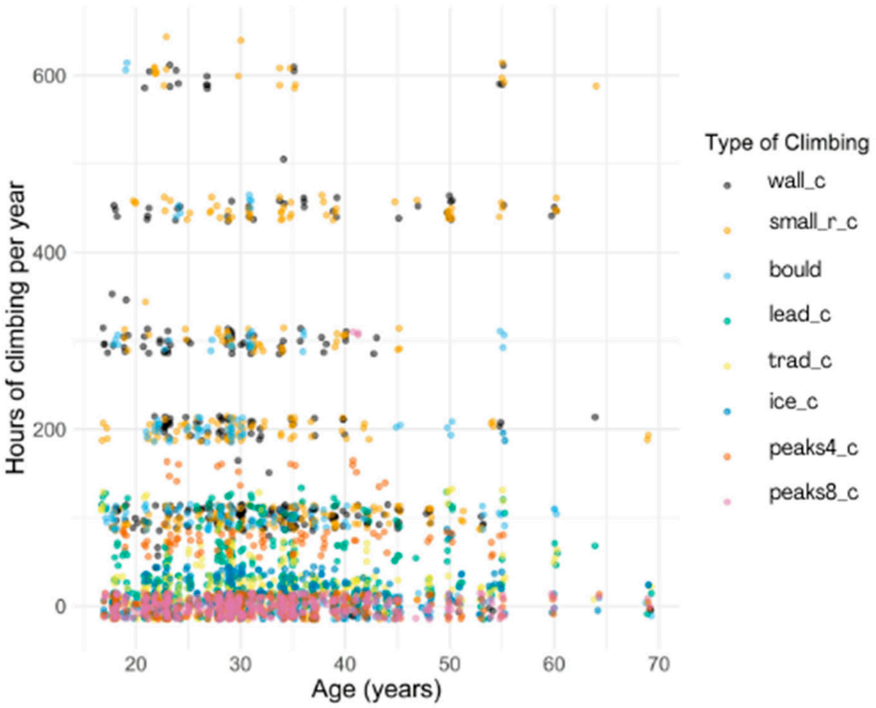
600
400
200
0
Hours of climbing per year
20
30
40
50
60
70
Age (years)
Type of Climbing
wall_c
small_r_c
bould
lead_c
trad_c
ice_c
peaks4_c
peaks8_c

(**a**)

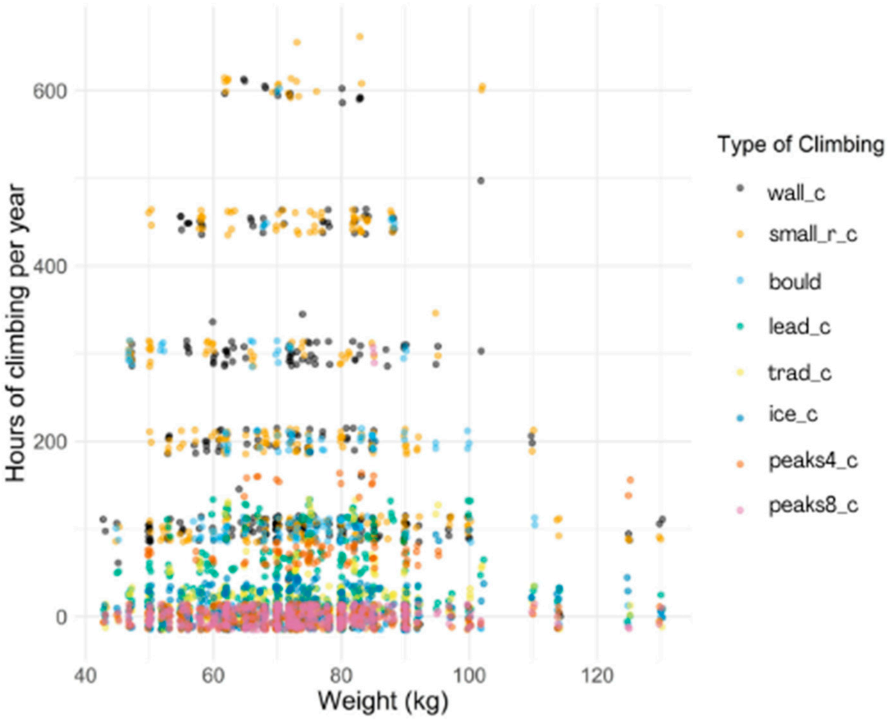
600
400
200
0
Hours of climbing per year
40
60
80
100
120
Weight (kg)
Type of Climbing
wall_c
small_r_c
bould
lead_c
trad_c
ice_c
peaks4_c
peaks8_c

(**b**)

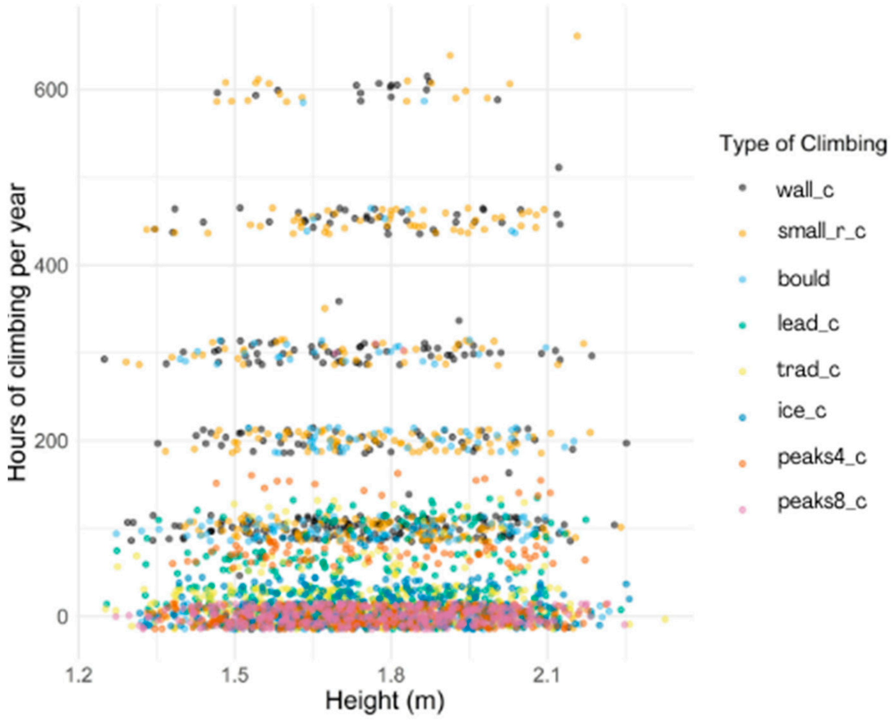

Hours of climbing per year
600
400
200
0
1.2
1.5
1.8
2.1
Height (m)
Type of Climbing
wall_c
small_r_c
bould
lead_c
trad_c
ice_c
peaks4_c
peaks8_c

(**c**)

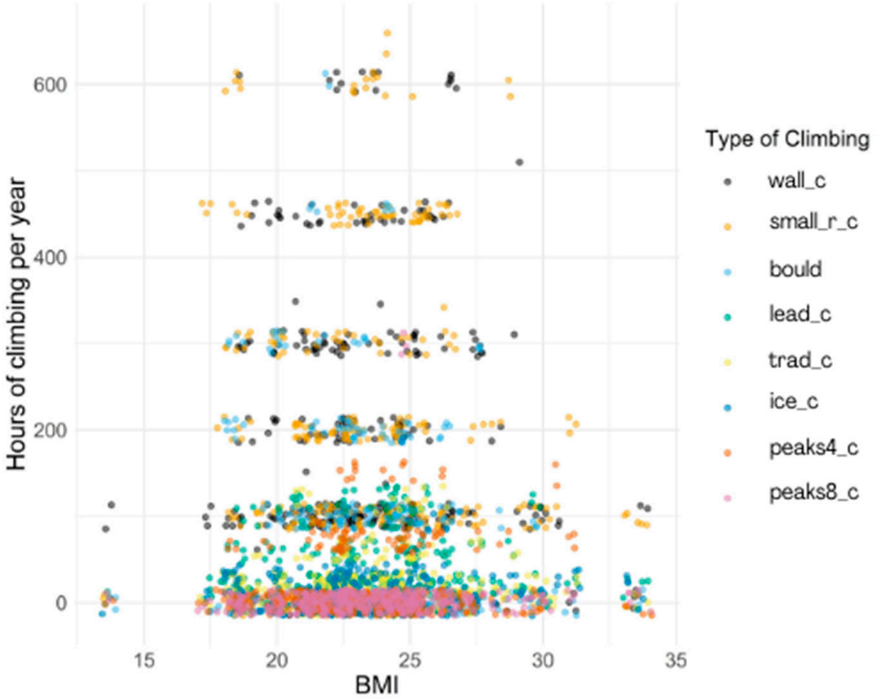

Hours of climbing per year
600
400
200
0
15
20
25
30
35
BMI
Type of Climbing
wall_c
small_r_c
bould
lead_c
trad_c
ice_c
peaks4_c
peaks8_c

(**d**)

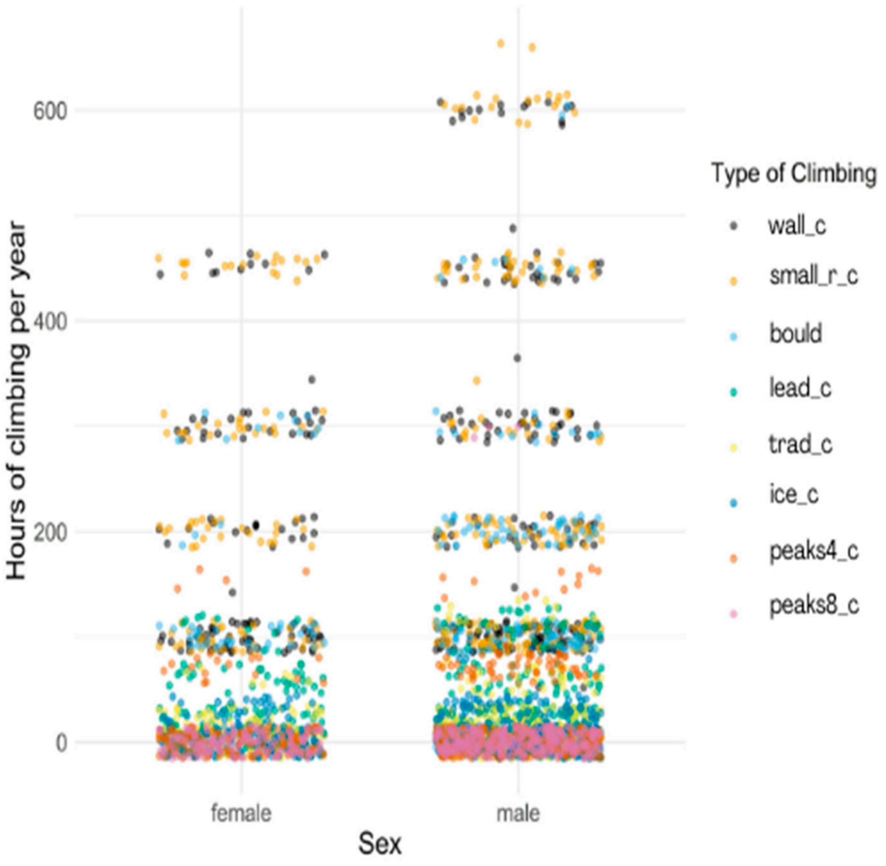

(**e**)

Supplement S3: Scatter plot of hours of climbing by: age (**a**), weight (**b**), height (**c**), BMI (**d**), sex (**e**) across different disciplines.
