# Supplementary material for: Analyzing Injury Patterns in Climbing: A Comprehensive Study of Risk Factors
Source: Sports (Basel). 2024 Feb 19;12(2):61. doi: 10.3390/sports12020061 (PMC10892067; doi:10.3390/sports12020061)
Supplement: Supplementary file 1 [file sports-12-00061-s001.zip › S4 ethic committee.pdf]

FAKULTNÍ NEMOCNICE OLOMOUC

Etická komise Fakultní nemocnice Olomouc a Lékařské fakulty UP v Olomouci
Zdravotníků 248/7, 779 00 Olomouc
předseda: MUDr.Jindřiška Burešová, tel: 588 443420, e-mail: jindriska.buresova@fnol.cz
tajemnice tel., fax 588442477, e-mail: iveta.sudolska@fnol.cz

STANOVISKO ETICKÉ KOMISE
Opinion of the Ethics Committee

Číslo jednací/*Reference number*: **173/23**

Název výzkumného projektu: Analyzing Injury Patterns in Climbing: A Comprehensive study of risk factors

Žadatel/*Applicant*: Mgr. Markéta Kovářová, Ústav normální anatomie, UP Olomouc

Datum doručení žádosti/*Date of submission of the Application Form*: 11.12.2023
Datum jednání EK /*Date of Ethics Committee´s session*: 11.12.2023

Vyjádření EK/ *Ethics Committe´s opinion*:
☒ EK vydala souhlasné stanovisko / *EC issued favourable opinion*
☒ EK vzala na vědomí / *Taken into account*

Seznam míst hodnocení s označením míst, ke kterým se EK vyjádřila jako místní EK a kde vykonává dohled/*List of clinical trial sites in the Czech Republic where EC has given its opinion and will perform supervision:*

Místo hodnocení/ Jméno zkoušejícího *Trial Site / Name of Investigator*	Místní EK *Local EC*	Adresa místní EK *Address*
Mgr. Markéta Kovářová, Ústav normální anatomie, UP Olomouc, Hněvotínská 3, 775 15 Olomouc	☒	EK FNOL

Seznam hodnocených dokumentů/*List of all submitted documents:*

**Název dokumentu, verze, datum** *Document title, version, date*	**Schváleno** /*Approved*		**Vzato na vědomí** / *Taken into account*	
	**ANO** *Yes*	**NE** *No*	**ANO** *Yes*	**NE** *No*
Žádost o projednání výzkumného projektu	☐	☐	☒	☐
Sylabus projektu	☒	☐	☐	☐
Informovaný souhlas vč. Informace pro pacienta	☒	☐	☐	☐
Strukturovaný životopis hlavního řešitele	☐	☐	☒	☐

Etická komise prohlašuje, že byla ustavena a pracuje podle jednacího řádu v souladu se správnou klinickou praxí (GCP) a platnými právními předpisy/*The Ethics Committee hereby declares that it was established and operates in accordance with its Rules of Procedure in compliance with Good Clinical Practice and valid legal regulations:*
☒ Ano/*Yes* ☐ Ne/*No*

Datum/*Date:* 11.12.2023

MUDr. Jindřiška Burešová
předsedkyně EK FNOL a LF UP
Chairman of the EC FNOL and LF UP

v. z.

ETHICS COMMITTEE
the University Hospital
and the Faculty Medicine
Palacky University in
OLOMOUC

Rozdělovník/*Distribution list:*
-Zadavatel
-EK
-Řešitel
1/1
