# Supplementary figures and images for: Analyzing Injury Patterns in Climbing: A Comprehensive Study of Risk Factors
Source: Sports (Basel). 2024 Feb 19;12(2):61. doi: 10.3390/sports12020061 (PMC10892067; doi:10.3390/sports12020061)

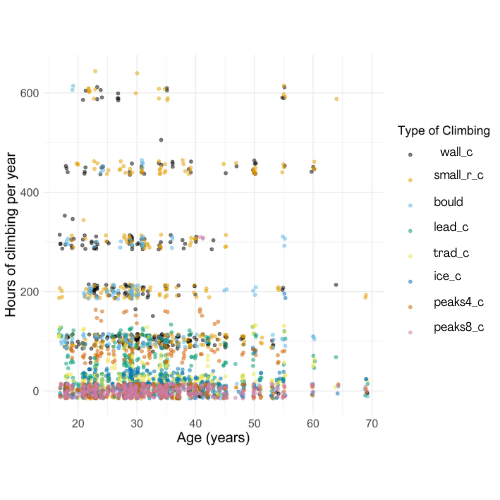

Supplement: Supplementary file 1 [file sports-12-00061-s001.zip › figure S3 a.png]

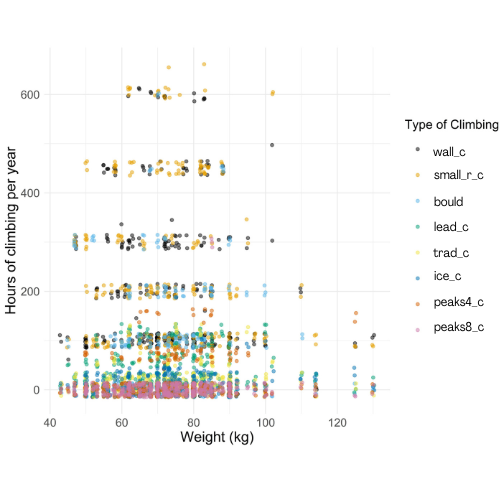

Supplement: Supplementary file 1 [file sports-12-00061-s001.zip › figure S3 b.png]

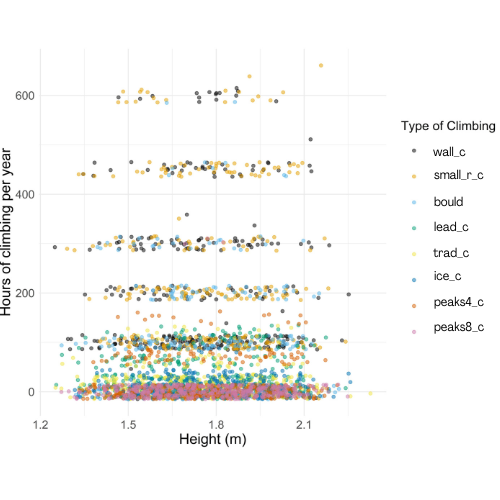

Supplement: Supplementary file 1 [file sports-12-00061-s001.zip › figure S3 c.png]

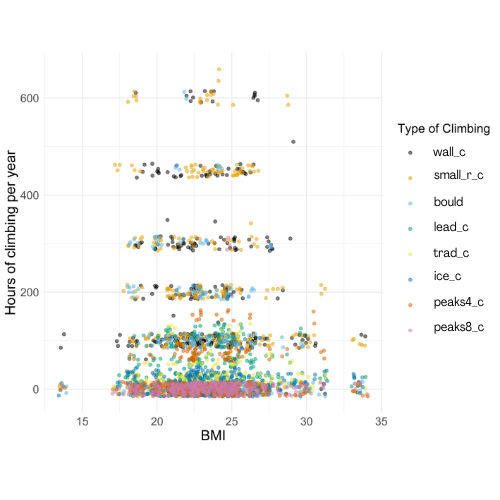

Supplement: Supplementary file 1 [file sports-12-00061-s001.zip › figure S3 d.png]

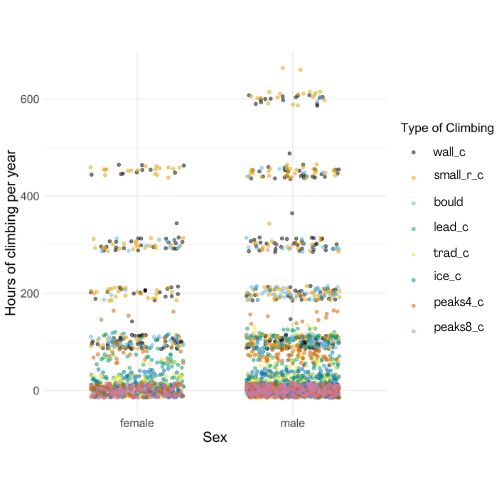

Supplement: Supplementary file 1 [file sports-12-00061-s001.zip › figure S3 e.png]

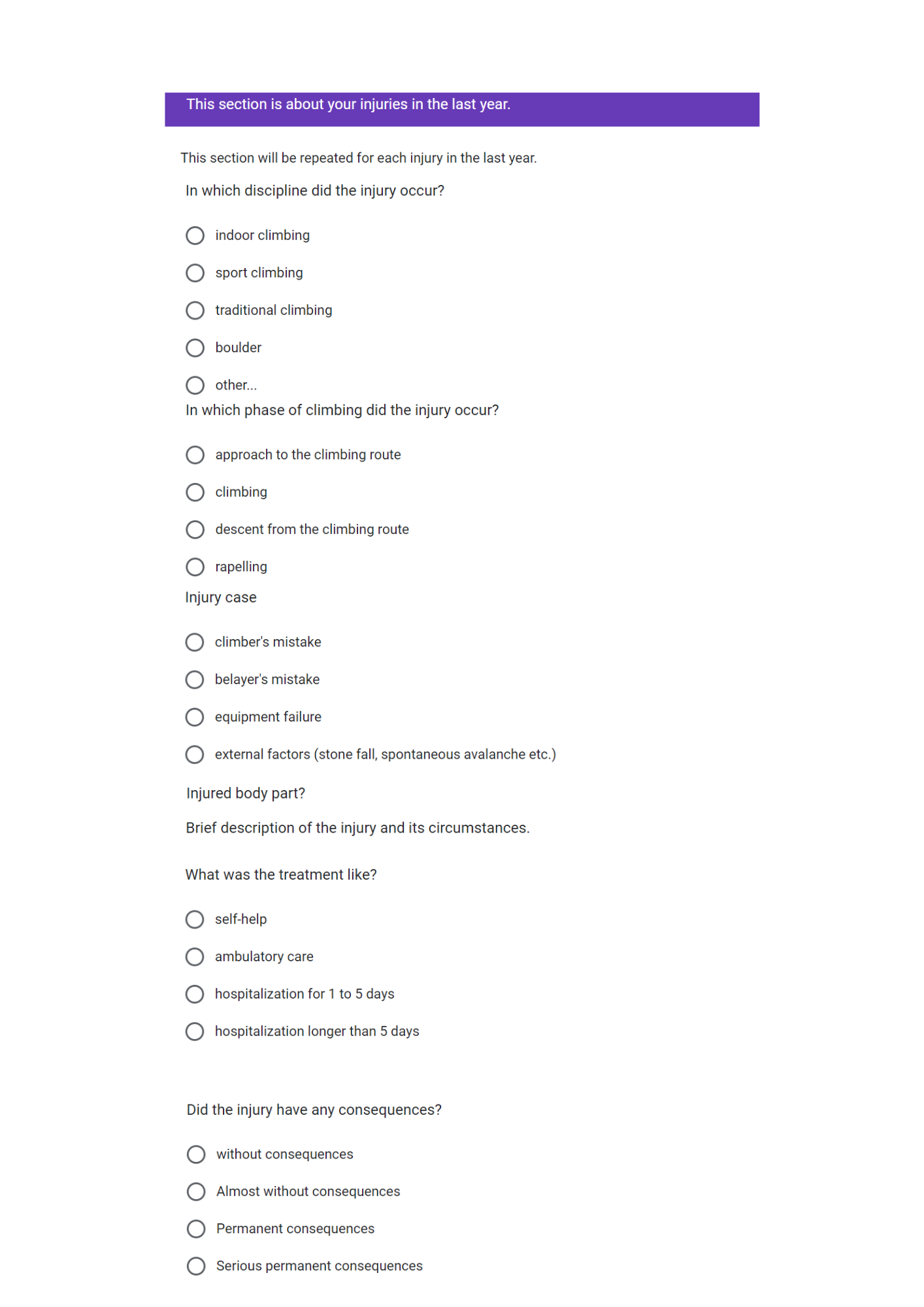

Supplement: Supplementary file 1 [file sports-12-00061-s001.zip › S1 a (first part).png]
